# 24-Methyl­lanosta-7,25-dien-3-one

**DOI:** 10.1107/S1600536810005118

**Published:** 2010-02-13

**Authors:** Nisar Hussain, Habib-ur-Rehman, Masood Parvez

**Affiliations:** a Department of Chemistry, University of Azad Jammu and Kashmir, Muzaffarabad 13100, Pakistan; b Department of Chemistry, University of Calgary, 2500 University Drive NW, Calgary, Alberta, Canada T2N 1N4

## Abstract

The title compound [systematic name: 17-(5,6-di­methyl­hept-6-en-2-yl)-4,4,10,13,14-penta­methyl-1,5,6,10,11,12,13,15,16,17-deca­hydro-2*H*-cyclo­penta­[α]phenanthren-3(4*H*,9*H*,14*H*)-one], C_31_H_50_O, is a triterpenoid which was isolated from *Skimmia laureola*. The three six-membered rings adopt chair, slightly distorted half-chair and distorted boat conformations, and the five-membered ring is in an envelope conformation. All the rings are *trans* fused. In the crystal structure, there is a weak C—H⋯O hydrogen bond.

## Related literature

For related structures, see: Hussain *et al.* (2009 ▶); Schun *et al.* (1986 ▶). For reference bond lengths, see: Allen *et al.* (1987 ▶). For puckering parameters, see: Cremer & Pople (1975 ▶).
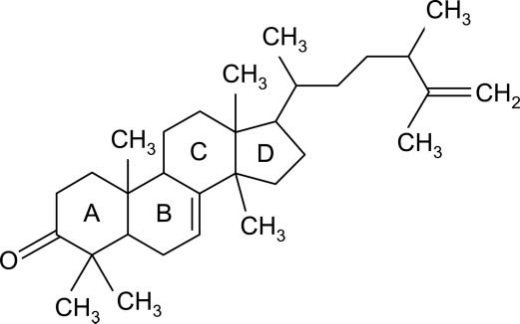


## Experimental

### 

#### Crystal data

C_31_H_50_O
*M* *_r_* = 438.71
Orthorhombic, 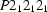

*a* = 6.7207 (1) Å
*b* = 19.4804 (5) Å
*c* = 20.5035 (5) Å
*V* = 2684.36 (10) Å^3^
*Z* = 4
Mo *K*α radiation
μ = 0.06 mm^−1^
*T* = 173 K
0.30 × 0.05 × 0.04 mm

#### Data collection

Nonius  diffractometer with Bruker APEXII CCD
Absorption correction: multi-scan (*SORTAV*; Blessing, 1997 ▶) *T*_min_ = 0.981, *T*_max_ = 0.997
6101 measured reflections
3485 independent reflections
2918 reflections with *I* > 2σ(*I*)
*R*_int_ = 0.036

#### Refinement

*R*[*F* ^2^ > 2σ(*F* ^2^)] = 0.055
*wR*(*F* ^2^) = 0.122
*S* = 1.15
3485 reflections
297 parameters
H-atom parameters constrained
Δρ_max_ = 0.21 e Å^−3^
Δρ_min_ = −0.20 e Å^−3^

### 

Data collection: *COLLECT* (Nonius, 1998 ▶); cell refinement: *HKL* *DENZO* (Otwinowski & Minor, 1997 ▶); data reduction: *SCALEPACK* (Otwinowski & Minor, 1997 ▶); program(s) used to solve structure: *SHELXS97* (Sheldrick, 2008 ▶); program(s) used to refine structure: *SHELXL97* (Sheldrick, 2008 ▶); molecular graphics: *ORTEP-3 for Windows* (Farrugia, 1997 ▶); software used to prepare material for publication: *SHELXL97*.

## Supplementary Material

Crystal structure: contains datablocks global, I. DOI: 10.1107/S1600536810005118/lh2983sup1.cif

Structure factors: contains datablocks I. DOI: 10.1107/S1600536810005118/lh2983Isup2.hkl

Additional supplementary materials:  crystallographic information; 3D view; checkCIF report

## Figures and Tables

**Table 1:** Hydrogen-bond geometry (Å, °)

*D*—H⋯*A*	*D*—H	H⋯*A*	*D*⋯*A*	*D*—H⋯*A*
C16—H16*A*⋯O1^i^	0.99	2.55	3.528 (4)	169

Symmetry code: (i) 
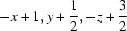
.

**Fig. 1.**
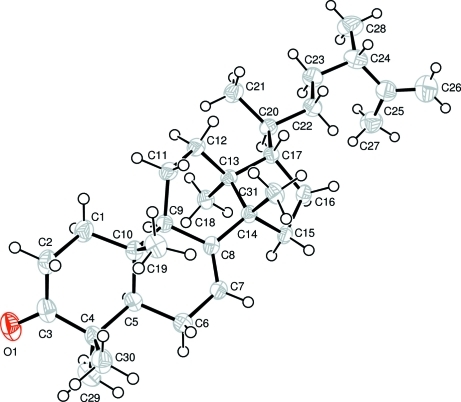
ORTEP-3 (Farrugia, 1997) drawing of (I) with displacement ellipsoids plotted at 50% probability level.

**Fig. 2.**
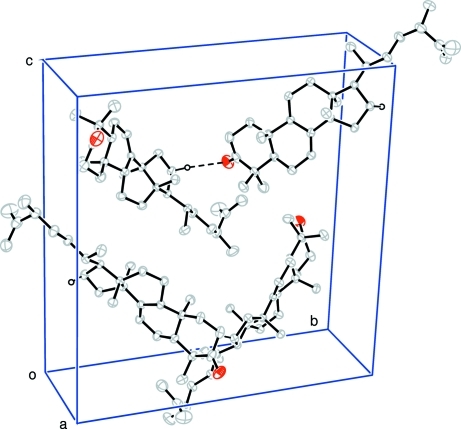
Unit cell packing of (I) showing non-classical hydrogen bonding interaction with dashed lines; H atoms not involved in H-bonds have been excluded for clarity.

## supplementary crystallographic information

### Comment

The methanol extract of *Skimmia laureola* affords a novel triterpene, *o*-methyl cyclolaudenol, the structure of which has been reported recently from our laboratory (Hussain *et al.* 2009). In this paper, we report the crystal structure of yet an other triterpene which has been isolated from *Skimmia laureola*, 17-(5,6-dimethylhept-6-en-2-yl)-4,4,10,13,14-pentamethyl- 1,5,6,10,11,12,13,15,16,17-decahydro-2*H*-cyclopenta[α]phenanthren- 3(4*H*,9H,14*H*)-one, (I).

The molecular structure of (I) is presented in Fig. 1. The molecule contains three six-membered rings, A, B and C and a five-membered ring, D. The ring A adopts a chair conformation. The rings B and C show disotortions due to the *trans*-fused ring D, exhibiting slightly distorted half-chair and distorted boat conformations, respectively. The puckering parameters (Cremer & Pople, 1975) for the rings A to C are: *Q* = 0.521 (3), 0.563 (3), 0.718 (3) Å, θ = 13.5 (3), 49.0 (3), 94.4 (2)° and φ = 27.6 (15), 319.3 (4), 89.9 (2)°, respectively. The ring D adopts a C14-envelope conformation. All rings are *trans* fused. The crystal structure of a very closely related compound, 24-methylene-25-methyltirucall-7-en-3-one, which is isomorphous with (I), has been reported (Schun *et al.*, 1986). The bond distances (Allen *et al.*, 1987) and angles in (I) are as expected. The structure is devoid of any classical hydrogen bonds. However, a non-classical hydrogen bonding interaction of the type C—H···O is present (Fig. 2 and Table 1).

### Experimental

The methanol extract of *Skimmia laureola* was subjected to silica-gel column chromatography. The column was eluted with increasing polarities of pet. ether/CHCl_3_. This afforded 4 fractions (PC1–PC4). The fraction PC3 (18 g) obtained by elution with 1 litre of pet. ether/CHCl_3_ (7.0:3.0) was subjected to the column chromatography. The column was successively eluted with 2 litre of pet. ether and 3 litre of pet. ether/CHCl_3_ (ranging from 9.0:1.0 to 7.0:3.0) to afford 7 fractions (PC3A–PC3G). The fraction PC3-G (1.4 g) obtained by elution of the column with 500 ml of pet. ether/CHCl_3_ (7.0:3.0) was further subjected to the column chromatography using 500 ml of pet. ether/CHCl_3_ (9.8:0.2) to afford the title triterpene, (I), as colourless crystals in needle form.

### Refinement

An absolute structure could not be established reliably becuase of insufficient anomalous scattering effects. Therefore, Friedel pairs (2616) were merged. All the H atoms were located from the difference Fourier maps and were included in the refinements at geometrically idealized positions with C—H distances = 0.95–1.00 Å, and *U*_iso_ = 1.5 and 1.2 times *U*_eq_ of the methyl and non-methyl C-atoms to which they were bonded. The final difference map was free of chemically significant features.

### Crystal data

**Table:**

C_31_H_50_O	*F*(000) = 976
*M**_r_* = 438.71	*D*_x_ = 1.086 Mg m^−^^3^
Orthorhombic, *P*2_1_2_1_2_1_	Mo *K*α radiation, λ = 0.71073 Å
Hall symbol: P 2ac 2ab	Cell parameters from 3435 reflections
*a* = 6.7207 (1) Å	θ = 1.0–27.5°
*b* = 19.4804 (5) Å	µ = 0.06 mm^−^^1^
*c* = 20.5035 (5) Å	*T* = 173 K
*V* = 2684.36 (10) Å^3^	Needle, colourless
*Z* = 4	0.30 × 0.05 × 0.04 mm

### Data collection

**Table:**

Nonius APEXII CCD diffractometer	3485 independent reflections
Radiation source: fine-focus sealed tube	2918 reflections with *I* > 2σ(*I*)
graphite	*R*_int_ = 0.036
φ and ω scans	θ_max_ = 27.5°, θ_min_ = 2.3°
Absorption correction: multi-scan (*SORTAV*; Blessing, 1997)	*h* = −8→8
*T*_min_ = 0.981, *T*_max_ = 0.997	*k* = −25→25
6101 measured reflections	*l* = −26→26

### Refinement

**Table:**

Refinement on *F*^2^	Primary atom site location: structure-invariant direct methods
Least-squares matrix: full	Secondary atom site location: difference Fourier map
*R*[*F*^2^ > 2σ(*F*^2^)] = 0.055	Hydrogen site location: inferred from neighbouring sites
*wR*(*F*^2^) = 0.122	H-atom parameters constrained
*S* = 1.15	*w* = 1/[σ^2^(*F*_o_^2^) + (0.0302*P*)^2^ + 1.38*P*] where *P* = (*F*_o_^2^ + 2*F*_c_^2^)/3
3485 reflections	(Δ/σ)_max_ < 0.001
297 parameters	Δρ_max_ = 0.21 e Å^−^^3^
0 restraints	Δρ_min_ = −0.19 e Å^−^^3^

### Special details

**Table:**

Geometry. All e.s.d.'s (except the e.s.d. in the dihedral angle between two l.s. planes) are estimated using the full covariance matrix. The cell e.s.d.'s are taken into account individually in the estimation of e.s.d.'s in distances, angles and torsion angles; correlations between e.s.d.'s in cell parameters are only used when they are defined by crystal symmetry. An approximate (isotropic) treatment of cell e.s.d.'s is used for estimating e.s.d.'s involving l.s. planes.
Refinement. Refinement of *F*^2^ against ALL reflections. The weighted *R*-factor *wR* and goodness of fit *S* are based on *F*^2^, conventional *R*-factors *R* are based on *F*, with *F* set to zero for negative *F*^2^. The threshold expression of *F*^2^ > σ(*F*^2^) is used only for calculating *R*-factors(gt) *etc*. and is not relevant to the choice of reflections for refinement. *R*-factors based on *F*^2^ are statistically about twice as large as those based on *F*, and *R*- factors based on ALL data will be even larger.An absolute structure could not be established reliably becuase of insufficient anomalous scattering effects. Therefore, Friedel pairs (2616) were merged.

### Fractional atomic coordinates and isotropic or equivalent isotropic displacement parameters (Å^2^)

**Table:**

	*x*	*y*	*z*	*U*_iso_*/*U*_eq_	
O1	0.8042 (3)	0.08030 (13)	0.84879 (12)	0.0529 (6)	
C1	0.5810 (5)	0.11757 (16)	0.70242 (16)	0.0381 (7)	
H1A	0.5754	0.0994	0.6574	0.046*	
H1B	0.6867	0.1529	0.7039	0.046*	
C2	0.6360 (5)	0.05905 (15)	0.74898 (15)	0.0409 (7)	
H2A	0.5329	0.0228	0.7472	0.049*	
H2B	0.7646	0.0386	0.7357	0.049*	
C3	0.6518 (5)	0.08664 (15)	0.81701 (16)	0.0371 (7)	
C4	0.4696 (4)	0.12362 (15)	0.84386 (14)	0.0329 (6)	
C5	0.3849 (4)	0.17454 (14)	0.79152 (13)	0.0282 (6)	
H5	0.4778	0.2146	0.7924	0.034*	
C6	0.1812 (5)	0.20449 (15)	0.81055 (14)	0.0351 (7)	
H6A	0.0845	0.1665	0.8159	0.042*	
H6B	0.1932	0.2284	0.8530	0.042*	
C7	0.1042 (4)	0.25387 (14)	0.76047 (13)	0.0308 (6)	
H7	−0.0055	0.2821	0.7720	0.037*	
C8	0.1798 (4)	0.26067 (13)	0.70088 (13)	0.0251 (6)	
C9	0.3595 (4)	0.21892 (13)	0.67906 (12)	0.0264 (6)	
H9	0.4789	0.2473	0.6900	0.032*	
C10	0.3811 (4)	0.15119 (14)	0.71894 (13)	0.0292 (6)	
C11	0.3635 (5)	0.20920 (14)	0.60485 (13)	0.0334 (7)	
H11A	0.2515	0.1790	0.5921	0.040*	
H11B	0.4886	0.1857	0.5927	0.040*	
C12	0.3484 (5)	0.27749 (14)	0.56601 (13)	0.0322 (6)	
H12A	0.4841	0.2918	0.5530	0.039*	
H12B	0.2717	0.2689	0.5256	0.039*	
C13	0.2496 (4)	0.33709 (13)	0.60346 (12)	0.0246 (5)	
C14	0.0865 (4)	0.30780 (13)	0.64965 (13)	0.0253 (5)	
C15	−0.0160 (4)	0.37378 (14)	0.67363 (13)	0.0291 (6)	
H15A	0.0609	0.3955	0.7093	0.035*	
H15B	−0.1525	0.3641	0.6893	0.035*	
C16	−0.0200 (4)	0.42041 (14)	0.61205 (13)	0.0292 (6)	
H16A	0.0215	0.4677	0.6234	0.035*	
H16B	−0.1559	0.4222	0.5936	0.035*	
C17	0.1276 (4)	0.38854 (13)	0.56168 (12)	0.0256 (6)	
H17	0.0464	0.3608	0.5305	0.031*	
C18	0.4052 (4)	0.37806 (14)	0.64288 (14)	0.0313 (6)	
H18A	0.3456	0.4210	0.6583	0.038*	
H18B	0.5197	0.3885	0.6150	0.038*	
H18C	0.4491	0.3507	0.6803	0.038*	
C19	0.2087 (5)	0.10171 (15)	0.70309 (16)	0.0376 (7)	
H19A	0.0817	0.1259	0.7078	0.045*	
H19B	0.2124	0.0627	0.7332	0.045*	
H19C	0.2225	0.0851	0.6582	0.045*	
C20	0.2346 (4)	0.44415 (14)	0.52139 (13)	0.0294 (6)	
H20	0.2982	0.4771	0.5525	0.035*	
C21	0.3977 (5)	0.41495 (16)	0.47722 (15)	0.0417 (7)	
H21A	0.4621	0.4525	0.4534	0.050*	
H21B	0.3386	0.3827	0.4461	0.050*	
H21C	0.4968	0.3910	0.5039	0.050*	
C22	0.0797 (5)	0.48390 (14)	0.48107 (14)	0.0339 (7)	
H22A	−0.0002	0.4505	0.4558	0.041*	
H22B	−0.0116	0.5078	0.5114	0.041*	
C23	0.1663 (5)	0.53685 (16)	0.43382 (15)	0.0412 (8)	
H23A	0.2550	0.5129	0.4027	0.049*	
H23B	0.2488	0.5697	0.4589	0.049*	
C24	0.0101 (6)	0.57747 (17)	0.39493 (15)	0.0454 (8)	
H24	−0.0784	0.5436	0.3725	0.054*	
C25	−0.1188 (6)	0.62273 (18)	0.43633 (17)	0.0471 (8)	
C26	−0.3137 (7)	0.6268 (3)	0.4248 (2)	0.0811 (14)	
H26A	−0.3941	0.6572	0.4497	0.097*	
H26B	−0.3722	0.5992	0.3917	0.097*	
C27	−0.0217 (6)	0.66514 (19)	0.48788 (17)	0.0543 (9)	
H27A	−0.1183	0.6979	0.5056	0.065*	
H27B	0.0908	0.6902	0.4690	0.065*	
H27C	0.0262	0.6352	0.5229	0.065*	
C28	0.1132 (7)	0.6206 (2)	0.34208 (17)	0.0600 (11)	
H28A	0.0124	0.6432	0.3150	0.072*	
H28B	0.1955	0.5907	0.3147	0.072*	
H28C	0.1975	0.6554	0.3628	0.072*	
C29	0.5309 (6)	0.16468 (18)	0.90441 (16)	0.0489 (9)	
H29A	0.4131	0.1864	0.9236	0.059*	
H29B	0.6271	0.2001	0.8919	0.059*	
H29C	0.5916	0.1337	0.9364	0.059*	
C30	0.3232 (5)	0.06653 (16)	0.86562 (17)	0.0420 (8)	
H30A	0.2018	0.0876	0.8830	0.050*	
H30B	0.3857	0.0383	0.8995	0.050*	
H30C	0.2894	0.0376	0.8281	0.050*	
C31	−0.0734 (4)	0.26625 (14)	0.61295 (14)	0.0322 (6)	
H31A	−0.1449	0.2965	0.5828	0.039*	
H31B	−0.1674	0.2466	0.6444	0.039*	
H31C	−0.0096	0.2292	0.5883	0.039*	

### Atomic displacement parameters (Å^2^)

**Table:**

	*U*^11^	*U*^22^	*U*^33^	*U*^12^	*U*^13^	*U*^23^
O1	0.0341 (12)	0.0587 (15)	0.0660 (16)	0.0023 (12)	−0.0095 (12)	0.0157 (13)
C1	0.0374 (17)	0.0334 (15)	0.0436 (18)	0.0087 (14)	0.0071 (15)	0.0081 (14)
C2	0.0351 (17)	0.0348 (15)	0.0529 (19)	0.0089 (14)	0.0042 (16)	0.0070 (14)
C3	0.0296 (16)	0.0305 (14)	0.0511 (17)	−0.0051 (13)	−0.0015 (14)	0.0173 (14)
C4	0.0299 (15)	0.0327 (14)	0.0362 (15)	−0.0002 (13)	−0.0024 (13)	0.0103 (12)
C5	0.0249 (14)	0.0276 (13)	0.0322 (14)	−0.0035 (12)	−0.0014 (12)	0.0056 (11)
C6	0.0352 (16)	0.0365 (15)	0.0336 (15)	0.0044 (14)	0.0031 (13)	0.0050 (12)
C7	0.0273 (14)	0.0334 (14)	0.0316 (14)	0.0039 (12)	0.0034 (13)	0.0025 (11)
C8	0.0216 (13)	0.0229 (12)	0.0308 (13)	−0.0022 (11)	−0.0018 (11)	−0.0032 (10)
C9	0.0243 (14)	0.0272 (13)	0.0277 (12)	0.0012 (11)	0.0000 (11)	0.0004 (11)
C10	0.0265 (14)	0.0275 (13)	0.0336 (14)	0.0013 (12)	0.0003 (12)	0.0020 (11)
C11	0.0396 (17)	0.0308 (14)	0.0298 (13)	0.0101 (14)	0.0041 (13)	−0.0005 (11)
C12	0.0348 (16)	0.0318 (14)	0.0301 (14)	0.0062 (13)	0.0036 (13)	0.0007 (12)
C13	0.0238 (13)	0.0256 (12)	0.0244 (12)	−0.0015 (11)	0.0012 (11)	−0.0016 (10)
C14	0.0213 (13)	0.0266 (12)	0.0280 (13)	−0.0010 (11)	0.0002 (11)	0.0013 (10)
C15	0.0225 (13)	0.0315 (14)	0.0333 (14)	0.0060 (12)	0.0037 (12)	0.0013 (12)
C16	0.0236 (13)	0.0293 (13)	0.0348 (14)	0.0027 (12)	0.0026 (12)	0.0023 (11)
C17	0.0242 (14)	0.0258 (12)	0.0268 (12)	0.0012 (11)	−0.0003 (11)	0.0018 (10)
C18	0.0254 (14)	0.0331 (14)	0.0356 (15)	−0.0031 (12)	−0.0029 (13)	−0.0001 (13)
C19	0.0395 (17)	0.0295 (14)	0.0439 (17)	−0.0045 (13)	−0.0063 (15)	−0.0005 (13)
C20	0.0293 (15)	0.0287 (13)	0.0302 (13)	−0.0028 (12)	0.0030 (12)	0.0009 (11)
C21	0.0418 (18)	0.0413 (16)	0.0419 (16)	0.0059 (16)	0.0115 (15)	0.0104 (14)
C22	0.0376 (17)	0.0299 (13)	0.0343 (15)	−0.0003 (13)	0.0017 (14)	0.0063 (12)
C23	0.0446 (19)	0.0379 (16)	0.0412 (17)	−0.0010 (15)	0.0044 (15)	0.0107 (14)
C24	0.060 (2)	0.0377 (16)	0.0381 (16)	−0.0077 (17)	−0.0018 (17)	0.0070 (14)
C25	0.049 (2)	0.0470 (18)	0.0452 (18)	0.0015 (17)	0.0032 (17)	0.0145 (16)
C26	0.054 (3)	0.114 (4)	0.075 (3)	0.006 (3)	−0.001 (2)	0.005 (3)
C27	0.062 (2)	0.0481 (19)	0.053 (2)	0.0064 (19)	0.004 (2)	0.0004 (17)
C28	0.080 (3)	0.054 (2)	0.0456 (19)	0.006 (2)	0.016 (2)	0.0181 (17)
C29	0.055 (2)	0.0503 (19)	0.0419 (18)	0.0037 (18)	−0.0130 (17)	0.0045 (15)
C30	0.0334 (16)	0.0398 (17)	0.0527 (19)	0.0008 (14)	0.0029 (15)	0.0161 (15)
C31	0.0278 (15)	0.0324 (14)	0.0365 (15)	−0.0066 (12)	−0.0071 (13)	0.0033 (12)

### Geometric parameters (Å, °)

**Table:**

O1—C3	1.220 (4)	C17—C20	1.540 (4)
C1—C2	1.532 (4)	C17—H17	1.0000
C1—C10	1.533 (4)	C18—H18A	0.9800
C1—H1A	0.9900	C18—H18B	0.9800
C1—H1B	0.9900	C18—H18C	0.9800
C2—C3	1.499 (4)	C19—H19A	0.9800
C2—H2A	0.9900	C19—H19B	0.9800
C2—H2B	0.9900	C19—H19C	0.9800
C3—C4	1.524 (4)	C20—C21	1.532 (4)
C4—C29	1.533 (4)	C20—C22	1.538 (4)
C4—C30	1.551 (4)	C20—H20	1.0000
C4—C5	1.568 (4)	C21—H21A	0.9800
C5—C6	1.539 (4)	C21—H21B	0.9800
C5—C10	1.556 (4)	C21—H21C	0.9800
C5—H5	1.0000	C22—C23	1.530 (4)
C6—C7	1.499 (4)	C22—H22A	0.9900
C6—H6A	0.9900	C22—H22B	0.9900
C6—H6B	0.9900	C23—C24	1.538 (5)
C7—C8	1.330 (4)	C23—H23A	0.9900
C7—H7	0.9500	C23—H23B	0.9900
C8—C9	1.523 (4)	C24—C25	1.499 (5)
C8—C14	1.530 (4)	C24—C28	1.536 (4)
C9—C11	1.534 (4)	C24—H24	1.0000
C9—C10	1.559 (4)	C25—C26	1.333 (6)
C9—H9	1.0000	C25—C27	1.492 (5)
C10—C19	1.542 (4)	C26—H26A	0.9500
C11—C12	1.554 (4)	C26—H26B	0.9500
C11—H11A	0.9900	C27—H27A	0.9800
C11—H11B	0.9900	C27—H27B	0.9800
C12—C13	1.542 (4)	C27—H27C	0.9800
C12—H12A	0.9900	C28—H28A	0.9800
C12—H12B	0.9900	C28—H28B	0.9800
C13—C18	1.544 (4)	C28—H28C	0.9800
C13—C17	1.552 (4)	C29—H29A	0.9800
C13—C14	1.557 (4)	C29—H29B	0.9800
C14—C15	1.539 (4)	C29—H29C	0.9800
C14—C31	1.541 (4)	C30—H30A	0.9800
C15—C16	1.556 (4)	C30—H30B	0.9800
C15—H15A	0.9900	C30—H30C	0.9800
C15—H15B	0.9900	C31—H31A	0.9800
C16—C17	1.561 (4)	C31—H31B	0.9800
C16—H16A	0.9900	C31—H31C	0.9800
C16—H16B	0.9900		
			
C2—C1—C10	113.1 (3)	H16A—C16—H16B	108.6
C2—C1—H1A	109.0	C20—C17—C13	120.2 (2)
C10—C1—H1A	109.0	C20—C17—C16	111.8 (2)
C2—C1—H1B	109.0	C13—C17—C16	103.1 (2)
C10—C1—H1B	109.0	C20—C17—H17	107.0
H1A—C1—H1B	107.8	C13—C17—H17	107.0
C3—C2—C1	109.3 (2)	C16—C17—H17	107.0
C3—C2—H2A	109.8	C13—C18—H18A	109.5
C1—C2—H2A	109.8	C13—C18—H18B	109.5
C3—C2—H2B	109.8	H18A—C18—H18B	109.5
C1—C2—H2B	109.8	C13—C18—H18C	109.5
H2A—C2—H2B	108.3	H18A—C18—H18C	109.5
O1—C3—C2	121.3 (3)	H18B—C18—H18C	109.5
O1—C3—C4	122.0 (3)	C10—C19—H19A	109.5
C2—C3—C4	116.7 (3)	C10—C19—H19B	109.5
C3—C4—C29	108.9 (3)	H19A—C19—H19B	109.5
C3—C4—C30	106.0 (2)	C10—C19—H19C	109.5
C29—C4—C30	108.2 (3)	H19A—C19—H19C	109.5
C3—C4—C5	110.1 (2)	H19B—C19—H19C	109.5
C29—C4—C5	108.8 (2)	C21—C20—C22	110.7 (2)
C30—C4—C5	114.8 (2)	C21—C20—C17	113.0 (2)
C6—C5—C10	109.8 (2)	C22—C20—C17	109.1 (2)
C6—C5—C4	112.9 (2)	C21—C20—H20	108.0
C10—C5—C4	118.4 (2)	C22—C20—H20	108.0
C6—C5—H5	104.8	C17—C20—H20	108.0
C10—C5—H5	104.8	C20—C21—H21A	109.5
C4—C5—H5	104.8	C20—C21—H21B	109.5
C7—C6—C5	112.1 (2)	H21A—C21—H21B	109.5
C7—C6—H6A	109.2	C20—C21—H21C	109.5
C5—C6—H6A	109.2	H21A—C21—H21C	109.5
C7—C6—H6B	109.2	H21B—C21—H21C	109.5
C5—C6—H6B	109.2	C23—C22—C20	115.0 (3)
H6A—C6—H6B	107.9	C23—C22—H22A	108.5
C8—C7—C6	124.2 (3)	C20—C22—H22A	108.5
C8—C7—H7	117.9	C23—C22—H22B	108.5
C6—C7—H7	117.9	C20—C22—H22B	108.5
C7—C8—C9	121.3 (2)	H22A—C22—H22B	107.5
C7—C8—C14	122.3 (2)	C22—C23—C24	114.5 (3)
C9—C8—C14	116.4 (2)	C22—C23—H23A	108.6
C8—C9—C11	111.8 (2)	C24—C23—H23A	108.6
C8—C9—C10	111.8 (2)	C22—C23—H23B	108.6
C11—C9—C10	114.5 (2)	C24—C23—H23B	108.6
C8—C9—H9	106.0	H23A—C23—H23B	107.6
C11—C9—H9	106.0	C25—C24—C28	109.8 (3)
C10—C9—H9	106.0	C25—C24—C23	113.8 (3)
C1—C10—C19	110.2 (2)	C28—C24—C23	109.8 (3)
C1—C10—C5	108.8 (2)	C25—C24—H24	107.7
C19—C10—C5	113.4 (2)	C28—C24—H24	107.7
C1—C10—C9	109.1 (2)	C23—C24—H24	107.7
C19—C10—C9	110.4 (2)	C26—C25—C27	121.5 (4)
C5—C10—C9	104.8 (2)	C26—C25—C24	120.1 (4)
C9—C11—C12	113.7 (2)	C27—C25—C24	118.3 (3)
C9—C11—H11A	108.8	C25—C26—H26A	120.0
C12—C11—H11A	108.8	C25—C26—H26B	120.0
C9—C11—H11B	108.8	H26A—C26—H26B	120.0
C12—C11—H11B	108.8	C25—C27—H27A	109.5
H11A—C11—H11B	107.7	C25—C27—H27B	109.5
C13—C12—C11	114.7 (2)	H27A—C27—H27B	109.5
C13—C12—H12A	108.6	C25—C27—H27C	109.5
C11—C12—H12A	108.6	H27A—C27—H27C	109.5
C13—C12—H12B	108.6	H27B—C27—H27C	109.5
C11—C12—H12B	108.6	C24—C28—H28A	109.5
H12A—C12—H12B	107.6	C24—C28—H28B	109.5
C12—C13—C18	111.0 (2)	H28A—C28—H28B	109.5
C12—C13—C17	116.0 (2)	C24—C28—H28C	109.5
C18—C13—C17	108.2 (2)	H28A—C28—H28C	109.5
C12—C13—C14	109.3 (2)	H28B—C28—H28C	109.5
C18—C13—C14	110.4 (2)	C4—C29—H29A	109.5
C17—C13—C14	101.6 (2)	C4—C29—H29B	109.5
C8—C14—C15	117.7 (2)	H29A—C29—H29B	109.5
C8—C14—C31	107.8 (2)	C4—C29—H29C	109.5
C15—C14—C31	106.4 (2)	H29A—C29—H29C	109.5
C8—C14—C13	110.4 (2)	H29B—C29—H29C	109.5
C15—C14—C13	101.7 (2)	C4—C30—H30A	109.5
C31—C14—C13	112.7 (2)	C4—C30—H30B	109.5
C14—C15—C16	103.6 (2)	H30A—C30—H30B	109.5
C14—C15—H15A	111.0	C4—C30—H30C	109.5
C16—C15—H15A	111.0	H30A—C30—H30C	109.5
C14—C15—H15B	111.0	H30B—C30—H30C	109.5
C16—C15—H15B	111.0	C14—C31—H31A	109.5
H15A—C15—H15B	109.0	C14—C31—H31B	109.5
C15—C16—C17	107.1 (2)	H31A—C31—H31B	109.5
C15—C16—H16A	110.3	C14—C31—H31C	109.5
C17—C16—H16A	110.3	H31A—C31—H31C	109.5
C15—C16—H16B	110.3	H31B—C31—H31C	109.5
C17—C16—H16B	110.3		
			
C10—C1—C2—C3	−60.6 (3)	C11—C12—C13—C17	−147.3 (2)
C1—C2—C3—O1	−123.0 (3)	C11—C12—C13—C14	−33.2 (3)
C1—C2—C3—C4	56.2 (3)	C7—C8—C14—C15	32.7 (4)
O1—C3—C4—C29	14.7 (4)	C9—C8—C14—C15	−150.2 (2)
C2—C3—C4—C29	−164.5 (2)	C7—C8—C14—C31	−87.6 (3)
O1—C3—C4—C30	−101.4 (3)	C9—C8—C14—C31	89.5 (3)
C2—C3—C4—C30	79.4 (3)	C7—C8—C14—C13	148.8 (3)
O1—C3—C4—C5	133.9 (3)	C9—C8—C14—C13	−34.0 (3)
C2—C3—C4—C5	−45.3 (3)	C12—C13—C14—C8	63.0 (3)
C3—C4—C5—C6	170.2 (2)	C18—C13—C14—C8	−59.3 (3)
C29—C4—C5—C6	−70.6 (3)	C17—C13—C14—C8	−173.9 (2)
C30—C4—C5—C6	50.7 (3)	C12—C13—C14—C15	−171.2 (2)
C3—C4—C5—C10	39.9 (3)	C18—C13—C14—C15	66.5 (3)
C29—C4—C5—C10	159.1 (3)	C17—C13—C14—C15	−48.2 (2)
C30—C4—C5—C10	−79.6 (3)	C12—C13—C14—C31	−57.7 (3)
C10—C5—C6—C7	−46.4 (3)	C18—C13—C14—C31	−180.0 (2)
C4—C5—C6—C7	179.1 (2)	C17—C13—C14—C31	65.4 (3)
C5—C6—C7—C8	13.1 (4)	C8—C14—C15—C16	159.4 (2)
C6—C7—C8—C9	−1.8 (4)	C31—C14—C15—C16	−79.6 (2)
C6—C7—C8—C14	175.2 (3)	C13—C14—C15—C16	38.6 (3)
C7—C8—C9—C11	154.0 (3)	C14—C15—C16—C17	−14.9 (3)
C14—C8—C9—C11	−23.2 (3)	C12—C13—C17—C20	−78.1 (3)
C7—C8—C9—C10	24.2 (4)	C18—C13—C17—C20	47.3 (3)
C14—C8—C9—C10	−153.0 (2)	C14—C13—C17—C20	163.5 (2)
C2—C1—C10—C19	−70.5 (3)	C12—C13—C17—C16	156.6 (2)
C2—C1—C10—C5	54.3 (3)	C18—C13—C17—C16	−78.0 (2)
C2—C1—C10—C9	168.1 (3)	C14—C13—C17—C16	38.2 (2)
C6—C5—C10—C1	−176.6 (2)	C15—C16—C17—C20	−145.2 (2)
C4—C5—C10—C1	−44.9 (3)	C15—C16—C17—C13	−14.6 (3)
C6—C5—C10—C19	−53.6 (3)	C13—C17—C20—C21	52.2 (3)
C4—C5—C10—C19	78.1 (3)	C16—C17—C20—C21	173.3 (2)
C6—C5—C10—C9	66.9 (3)	C13—C17—C20—C22	175.7 (2)
C4—C5—C10—C9	−161.5 (2)	C16—C17—C20—C22	−63.2 (3)
C8—C9—C10—C1	−171.0 (2)	C21—C20—C22—C23	−49.9 (3)
C11—C9—C10—C1	60.5 (3)	C17—C20—C22—C23	−174.8 (2)
C8—C9—C10—C19	67.7 (3)	C20—C22—C23—C24	−178.6 (3)
C11—C9—C10—C19	−60.7 (3)	C22—C23—C24—C25	65.2 (4)
C8—C9—C10—C5	−54.7 (3)	C22—C23—C24—C28	−171.3 (3)
C11—C9—C10—C5	176.9 (2)	C28—C24—C25—C26	98.2 (5)
C8—C9—C11—C12	53.3 (3)	C23—C24—C25—C26	−138.3 (4)
C10—C9—C11—C12	−178.2 (2)	C28—C24—C25—C27	−78.8 (4)
C9—C11—C12—C13	−23.7 (4)	C23—C24—C25—C27	44.7 (4)
C11—C12—C13—C18	88.7 (3)		

### Hydrogen-bond geometry (Å, °)

**Table:**

*D*—H···*A*	*D*—H	H···*A*	*D*···*A*	*D*—H···*A*
C16—H16A···O1^i^	0.99	2.55	3.528 (4)	169

Symmetry codes: (i) −*x*+1, *y*+1/2, −*z*+3/2.
